# Diagnosis of Injuries of the Shoulder-Joint

**Published:** 1856-11

**Authors:** 


### *Diagnosis of Injuries of the Shoulder-Joint.*

In our last number we reported a case, which illustrated the difficulties to be encountered in forming a correct diagnosis in injuries of the shoulder-joint. In connection with this subject, we present to our readers, the confession of a distinguished French surgeon, M. Lenoir. It was elicited by the discussion which took place on the 13th of October, 1852, at a meeting of the Societe de Chirurgie. We translate his remarks, as published in the *Bulletin de la Societe de Chirurgie*, tom. iii, p. 180.

REMARKS OF M. LENOIR.—You are aware that we have two kinds of fracture in this region. One, and that the most common through the surgical neck; the other, but rarely observed, through the anatomical neck. Now I admit nearly all that has been said about the facility of detecting a fracture through the surgical neck, and the value of the proceeding adopted by M. Richet, for reducing the dislocated head of the bone. In this accident the upper fragments are a certain volume, and something tangible to aid us in our exploration and reduction. But I deny that a fracture through the anatomical neck may be so readily detected. I deny that, in such a case, the head of the upper fragment can be grasped with facility, beneath the soft parts which envelop the shoulder, and I assert the impossibility of returning, by lateral pressure, the luxated bone into the glenoid cavity. I base my assertion upon two cases, which I find recorded in the annals of science, and upon one which came under my own observation. The first of these occurred in the practice of my old master (Houselot *pere*,) and formed the basis of the excellent memoirs published by Delpech, in his *Chirurge Clinique de Montpelier.* The specimen should now be in the Museum of the *Val-de-Grace*, where it was deposited by Houselot. The patient was a hemiplegic old man, who fell out of bed, and died from the effects of the injury at the end of twelve days. The head of the bone was thrown backwards into the infra spinous space. In this case the existence of the fracture could not be detected during life. The second case is reported by Sir Astley Cooper, in his excellent Treatice on Fractures and Dislocations. The fracture was intra capsular, and the head of the bone was thrown in front of the scapula, to which it had contracted, as shown by the autopsy, osseous adhesions. The patient died several days after the accident. At the time, she was treated by Mr. Lucus one of the surgeons of Guy's Hospital, who failed to detect the fracture. The third case occurred in my own practice. A woman æt. 88, by a fall on her shoulder, fractured the anatomical neck of the humerus. She died some five or six months after the accident, and at the autopsy, I found that the head of the bone had been thrown from the glenoid cavity, through a large rent in the capsular ligament, and that it rested, by its uneven surface, on the brachial plexus, its smooth and cartilaginous surface presented anteriorly, so that it had been thrown in a direction from above downwards.

Here, then, we have three cases of fracture of the anatomical neck of the humerus, complicated with a luxation which had been overlooked. Could this have been the result of inattention, or want of skill, on the part of the observers? Or, was it not owing to the fact, that, if not impossible, it is exceedingly difficult a diagnosis in these cases? A single glance at the symptoms by which such accidents may be characterized, will aid us in determining this point. In the first place they are produced only by extreme violence applied almost exclusively, to the stump of the shoulder; such violence always gives rise to a considerable extravasation of blood, tumefaction, and stiffness of the contused muscles; all of which circumstances are unfavorable to a thorough examination of a deeply seated fracture. Again, the deformity by which this accident is attended, is scarcely perceptible. Indeed, when the head of the humerus is separated from the tubercles, the upper fragment still presents a certain volume, and if it remain in the glenoid cavity, the stump of the shoulder will be but little depressed. I ask, then, is it possible for the most attentive surgeon to detect this slight depression, while the soft parts are still swolen? Crepitus, a pathognomonic symptom of fracture, is here absent or it may be indistinct, when the uneven surface of the lower fragment is brought in contact with the smooth, polished surface of the glenoid cavity. But the sensation thus produced is calculated rather to lead the surgeon to suppose, that, instead of a fracture, he has only a contussion to treat. Beside the symptoms already enumerated, we have the presence of the head of the bone in the vicinity of the glenoid cavity; if detected of course it will be the most positive test of the fracture and the luxation; but the tumefaction remaining for days after the accident will interfere with our examination, and, as the head of the bone is but a small size, may it not be so situated as to render it impossible for the most experienced surgeon to discover it? For these reasous, I maintain, that, in these cases, errors in diagnosis must often be committed.—*Western Lancet.*

